# What is the clinical and ethical importance of incidental abnormalities found by knee MRI?

**DOI:** 10.1186/ar2371

**Published:** 2008-02-05

**Authors:** Rebecca Grainger, Stephen Stuckey, Richard O'Sullivan, Susan R Davis, Peter R Ebeling, Anita E Wluka

**Affiliations:** 1 Department of Medicine, Wellington School of Medicine, University of Otago, 23A Mein St, Newtown, Wellington South 6021, New Zealand; 2 Malaghan Institute of Medical Research, Kelburn Parade, Wellington 6012, New Zealand; 3 Department of Diagnostic Imaging, Southern Health, Clayton Road, Clayton, Victoria, 3168, Australia; 4 MRI Unit, Epworth Hospital, 89 Bridge St, Richmond Victoria, 3121, Australia; 5 National Health and Medical Research Council of Australia Centre of Clinical Research Excellence for the Study of Women's Health Program, Department of Medicine, Monash University Medical School, Alfred Hospital, Melbourne, Victoria, 3004, Australia; 6 Departments of Medicine (RMH/WH) and Endocrinology, University of Melbourne, Western Hospital, University of Melbourne, Cnr Marian and Eleanor Streets, Footscray, Victoria, 3011, Australia; 7 Baker Heart Research Institute, AMREP, Commercial Road, Melbourne, Victoria, 3004, Australia; 8 Department of Epidemiology and Preventive Medicine, Monash University, Alfred Hospital, Commercial Road, Melbourne, Victoria, 3004, Australia

## Abstract

**Introduction:**

Magnetic resonance imaging (MRI) is increasingly used to examine joints for research purposes. It may detect both suspected and unsuspected abnormalities. This raises both clinical and ethical issues, especially when incidental abnormalities are detected. The prevalence of incidental, potentially clinically significant abnormalities identified by MRI and their clinical significance in a population undergoing knee MRI in research studies are unknown.

**Methods:**

We examined the prevalence of such lesions in healthy asymptomatic adults and those with symptomatic knee osteoarthritis (OA) undergoing knee MRI with limited sequences for the purpose of research. The MRI findings in 601 asymptomatic subjects and 132 with knee OA who underwent at least one limited knee MRI scan for cartilage volume measurement were examined by an MRI radiologist for the presence of potentially clinically significant abnormalities.

**Results:**

These were present in 2.3% of healthy and 2.3% of OA subjects. All required further investigation to exclude non-benign disease, including four with bone marrow expansion (0.7%), requiring further investigation and management. A single potentially life-threatening lesion, a myeloma lesion, was identified in a subject with symptomatic knee OA on their second MRI scan in a longitudinal study.

**Conclusion:**

As musculoskeletal MRI is increasingly used clinically and for research purposes, the potential for detecting unsuspected abnormalities that require further investigation should be recognized. Incorporating a system to detect these, to characterize unexpected findings, and to facilitate appropriate medical follow-up when designing studies using this technology should be considered ethical research practice.

## Introduction

Magnetic resonance imaging (MRI) has enhanced our ability to examine patients non-invasively. This allows us for the first time to examine factors that affect the earliest structural changes of osteoarthritis (OA) in healthy asymptomatic people prior to the onset of clinical and radiographic disease without risk to the subject. MRI enables the researcher to visualize and quantitate the changes in articular cartilage, the menisci, and other periarticular structures non-invasively [1,2], unlike other imaging modalities that do not image all structures equally. MRI has the additional advantage over radiography and computed tomography of not exposing subjects to ionizing radiation. This is a significant ethical benefit in longitudinal studies that require repetitive imaging of healthy subjects. MRI is thus being used increasingly for the imaging of joints for both clinical and research purposes [3,4].

However, these benefits come at a cost: MRI use in healthy populations will detect unsuspected abnormalities or incidental findings. In the non-clinical research setting, any abnormality, even if finally diagnosed as benign, is a matter of medical and ethical concern: the researcher has a duty of care to the study participant [5]. Although this problem is well documented in other clinical areas such as neurology, in which MRI is widely used [6-8], it has not been addressed by the musculoskeletal community. When a lesion is identified in a study MRI scan, this will incur additional costs for investigation and clinical management which should be anticipated and incorporated into study design [7]. However, without any prevalence data, the cost of this is difficult to predict. This is likely to be especially relevant in large studies examining healthy asymptomatic participants. For example, the Osteoarthritis Initiative is a large study initiated to examine risk factors for OA and its progression [4]. It is a public-private partnership between the National Institutes of Health (Bethesda, MD, USA) and industry planned to facilitate the identification of biomarkers for OA [4]. In this study, 5,000 subjects who are healthy and asymptomatic or who have early knee OA will undergo repeated MRI of their knees.

When MRI is performed within a study, the researcher performing the measurements may not be an MRI radiologist: they may be unable to detect abnormalities. Thus, the decision must be made whether to require that an MRI radiologist or other suitably trained specialist review all the images or just the baseline images in a longitudinal study. This will also add to the cost of the research study.

Despite the increasing use of MRI in this manner, no information on the prevalence of such lesions found incidentally on knee MRI performed on healthy subjects is available. We describe the prevalence of potentially clinically significant bone lesions found on MRI in healthy subjects and in those with knee OA who underwent MRI for research.

## Materials and methods

### Patients

All 601 healthy subjects and 132 subjects with knee OA who were more than 20 years old and who had undergone MRI assessment of their dominant or symptomatic knee (in subjects with knee OA) in studies of knee cartilage in our institution were identified. Information regarding age and gender was obtained for all subjects. All participants had given informed consent for participation in studies approved by the ethics committee of the Alfred Hospital in Melbourne, Australia [1,9-12]. Subjects had been recruited through advertising in newspapers, sporting clubs, and the hospital staff association. Subjects were excluded if any form of arthritis other than OA was present, including evidence of chondrocalcinosis on plain radiographs. Subjects were excluded if they had a contraindication to MRI (for example, pacemaker, cerebral aneurysm clip, cochlear implant, presence of shrapnel in strategic locations, metal in the eye, and claustrophobia), hemiparesis of either lower limb, or significant knee pain. Each subject had an MRI scan performed on their dominant knee (defined as the lower limb they step off from when walking). In the case of subjects with knee OA, the symptomatic knee was imaged, unless both knees were symptomatic, in which case the knee with the less severe radiographic OA was used to minimize drop-out for joint replacement in longitudinal study.

### Magnetic resonance imaging measurements

All knees were imaged in the sagittal plane on one of two 1.5-T whole-body magnetic resonance units, a General Electric Signa Advantage Hi Speed (GE Medical Systems, Milwaukee, WI, USA), using a commercial receive-only extremity coil, or a Phillips Intera 1.5T (Philips Medical Systems, Best, The Netherlands), using a commercial transmit-and-receive knee coil. The following sequences and parameters were used: (a) a T_1_-weighted fat-suppressed three-dimensional gradient echo acquisition in the steady state; flip angle 55 degrees; repetition time 58 milliseconds; echo time 12 milliseconds; field of view 16 cm; 60 partitions; 512 × 196 matrix; one acquisition, time 11 minutes 17 seconds; sagittal images were obtained at a partition thickness of 1.5 mm and an in-plane resolution of 0.31 × 0.82 (512 × 196 pixels) (all subjects); and (b) a coronal T_2_-weighted fat-saturated acquisition (repetition time 2,526 milliseconds; echo time 40 milliseconds) with a slice thickness of 3 mm, a 0.3-mm interslice gap, 3 excitations, a field of view of 14 cm, and a matrix of 256 × 128 pixels (516 subjects). At least one experienced clinical MRI radiologist reviewed each study MRI scan to determine whether there were any potentially clinically significant abnormalities. A clinically significant abnormality was one deemed by the experienced radiologist to require further clarification through either further imaging or clinical review.

## Results

Six hundred one healthy pain-free subjects, including 448 (74.5%) women, were imaged at least once, and 85 subjects were imaged on two occasions (Table 1). Of these, 520 (87%) were imaged on the Philips MRI scan and 81 (13%) on the GE MRI scan. One hundred thirty-two subjects with knee OA were imaged on at least one occasion, and 123 were imaged on at least two occasions. The mean age of healthy participants was 56.1 years (standard deviation [SD] 7.3). The mean age of OA participants was 63.6 years (SD 10.1). Four hundred seventy-seven subjects underwent scans using both imaging sequences, and the remainder (including all subjects with knee OA and all who underwent two scans) underwent only the T_1_-weighted scan sequence.

**Table 1:** Participant demographics

	Healthy subjects *N *= 601	Osteoarthritis subjects *N *= 132
Age in years, mean (standard deviation)	56.1 (7.3)	63.1 (10.5)
Females, number (percentage)	448 (64.5%)	72 (54.5%)
Repeat imaging for research purposes, number (percentage)	85 (14.1%)	123 (93.1%)

In healthy asymptomatic subjects, the prevalence of incidental abnormalities identified on the baseline scans was 2.3%. The lesions identified on initial scanning which were considered worthy of clarification were eventually identified as benign enchondroma (5), atypical Baker cyst (1), fibrous cortical defect (1), ganglion (3), and marrow hyperplasia (4). Whilst the Baker cyst was likely related to asymptomatic knee OA, it was atypical in that it pointed superiorly and laterally: typical Baker cysts point inferiorly and medially. Multiplanar MRI was required to ensure that this was not a parameniscal cyst, ganglion, or a cystic tumour, although these are less likely. Details of the abnormalities identified in healthy subjects and subject characteristics, including investigations (both clinical and radiological) required to more completely characterize the abnormality and exclude other significant pathologies, are presented in Table 2.

**Table 2:** Lesions identified on baseline scans in 2.3% of healthy subjects (14 of 601)

Lesion	Gender	Age in years	Outcome (investigation: final diagnosis/diagnoses)
Enchondroma	Female	54	Panel review: enchondroma
Enchondroma	Female	57	Panel review: enchondroma
Intramedullary lesion	Female	56	Panel review: benign enchondroma
Enchondroma	Female	61	X-ray, multi-slice computed tomography, panel review: lobulated enchondroma
Enchondroma	Male	72	X-ray, panel review: enchondroma
Marrow hyperplasia	Female	50	Physician review: haemoglobin 13.2 g/dL, blood donor, no additional reason for marrow hyperplasia
Marrow hyperplasia	Female	52	Haematological review: known anemia secondary to bleeding
Marrow hyperplasia	Female	54	Physician review: full blood examination stable over 2 years, no reason for marrow hyperplasia identified
Marrow hyperplasia	Female	60	Haematological review: anemia identified and treated
Ganglion	Male	73	Orthopedic surgeon and panel review: neuroma or soft tissue lesion identified. Ganglion likely. Differential diagnoses: haemangioma. Meniscal tear, chondromalacia patellae
Geode	Male	66	X-ray, MRI: medial meniscal tear, cartilage loss, medial femoral condyle and patellar cartilage, reactive marrow oedema, infra-patellar bursitis, osseous ganglion
Fibrous cortical defect	Female	52	X-ray, MRI: healed fibrous cortical defect
Baker cyst, atypical	Male	60	MRI: grade IV chondromalacia patella, medial meniscal tear, atypical Baker cyst (posteromedial joint line)
Ganglion	Female	63	Panel review: ganglion

MRI, magnetic resonance imaging.

In subjects with knee OA, the prevalence of lesions was approximately 2.3%. By means of the clinical and imaging investigations shown (Table 3), the lesions identified on initial MRI were identified as benign enchondromata (2) and an osteochondroma (1).

**Table 3:** Lesions identified on baseline scans in 2.3% of osteoarthritis subjects (3 of 132)

Lesion	Gender	Age in years	Outcome (investigation: final diagnosis)
Osteochondroma	Male	48	MRI, orthopedic review: osteochondroma
Enchondroma with endosteal remodeling	Male	57	X-ray, CT, DMSA, MRI, orthopedic panel review: enchondroma
Enchondroma	Female	63	CT, MRI, X-ray, DMSA, orthopedic panel review: enchondroma

CT, computed tomography; DMSA, technetium-99m pentavalent dimercaptosuccinic acid scintigraphy; MRI, magnetic resonance imaging.

In subjects who underwent more than one MRI scan, two additional abnormalities were identified in the follow-up scan (but not on the baseline scan): an osseous myeloma lesion (which had been previously diagnosed) and an enchondroma. Multiple myeloma had been diagnosed prior to the follow-up MRI in the affected participant and thus required no further investigation. The subject with the enchondroma identified only on the follow-up scan underwent a complete clinical MRI, which confirmed this diagnosis.

## Discussion

Our data suggest that MRI use in both healthy populations and those with knee OA will detect a significant number of incidental lesions, some of which are clinically significant and will require further imaging and clinical management. Furthermore, new lesions may develop during longitudinal studies, proscribing ongoing surveillance to ensure that these lesions are detected. Whilst the incidence of life-threatening lesions identified in musculoskeletal imaging is low, the onus is on the investigator to ensure that any abnormalities present are identified when imaging is performed for research purposes.

This is the first study to examine the prevalence of unsuspected, potentially clinically significant abnormalities identified by either musculoskeletal MRI or another imaging modality in a healthy population. Previous studies have reported case series of all identified bone and cartilaginous tumours identified by clinically indicated plain radiographs for non-musculoskeletal indications, such as sinus radiography [13]. In contrast, many of our participants were asymptomatic.

This study is limited in that subjects underwent diagnostic imaging using more limited sequences than would be used in a clinically indicated scan and an abnormality was defined as one a trained MRI radiologist deemed to require further investigation. Although use of more extensive sequences in the studies may have better characterized the identified abnormalities, they may also have identified additional abnormalities, so that the true prevalence of such lesions may be underestimated. However, further sequences would be neither financially viable nor optimal for research purposes although this may reduce the need for contacting the subjects to perform further investigation to confirm the nature of detected lesions. Conversely, it is possible that we have overestimated the prevalence of incidental findings in an asymptomatic population since the 'healthy' participants may self-select on the basis of knee pain or prior trauma. However, since we excluded subjects experiencing significant pain, requiring therapy, and those with a history of significant knee trauma, this is unlikely: these subjects would not have otherwise undergone knee imaging. Subjects with knee OA had already undergone radiography for study inclusion, which had not identified these lesions. It might be expected that the prevalence of lesions in studies of symptomatic OA will be higher since, for study inclusion, participants are often required to have knee pain. It is possible that pain may not be due to knee OA but to a coexisting, new, alternative pathology. Although we did not detect a difference between studies of healthy subjects and those involving participants with knee OA, our numbers were small and our power to detect this was limited. Indeed, our numbers were small and are able to give only an estimate of the prevalence of such lesions; a much larger study would be required to give a more precise prevalence estimate. However, this is the first study in this area.

Our findings raise issues for the planning of studies and ethical issues related to the obligation of the physician-researcher to study participants [5]. In our studies, we acquire limited MRI sequences, which reduce the cost of research. These scans are incomplete for diagnostic purposes but are adequate to indicate abnormality. Technicians trained in the measurement and scoring of structural change read the MRI scans: they are not usually trained in musculoskeletal radiology or MRI beyond the measurements they perform. The researcher is obliged to put the health of the participant above the study, so that any potential abnormality is fully investigated [5]. The implications of this are that, in addition to the study measurements being made, all images should be reviewed by a suitably trained specialist to ensure that no significant abnormality is present and that this should be factored into the study, as has been our practice. Where, as in Australia, there is universal health care, there is a facility to examine abnormalities appropriately. Thus, where a possible bone or soft tissue tumour is identified, the images are reviewed by a group of musculoskeletal radiologists, orthopedic surgeons, and pathologists who direct the researchers as to which further investigations, if any, are required [14]. If the findings are non-orthopedic, the participant is contacted and reviewed by a study rheumatologist to ensure appropriate medical follow-up. We arrange imaging and specialist review, where required, in a timely fashion to reduce participant distress. To facilitate appropriate follow-up, we request consent at study entry from participants to contact their primary care physician (and contact details) should there be unexpected study findings. This procedure has been approved by all of the relevant institutional human research and ethics committees overseeing our work.

These issues are not inconsequential and may add to the cost of research. Whilst the incidence of clinically significant abnormalities is low, with only one potentially life-threatening abnormality identified in this study, the implications of failing to identify one (if present) are significant. The distal femur and tibia are sites where bone and cartilage tumours are more commonly seen than at other sites [15]. Similarly, these bones may be affected by red marrow expansion (due to anemia, vitamin deficiency, malignancy, and so on), which may also have significant clinical ramifications. In contrast, other imaged areas, such as the hand and wrist imaged for rheumatoid arthritis, may be less likely to show these abnormalities [15]. Although 28% of enchondromas are seen in the hand, these are benign lesions [15].

These data suggest the need for a system to be in place for studies using imaging techniques in order to ensure that potentially clinically significant lesions, incidental to the purpose of the imaging, are not missed. There may be a number of ways of providing this function apart from review by a musculoskeletal radiologist or other suitably trained specialist. Whilst it may be possible to train an experienced non-radiologist to screen images to identify those that require further investigation, the potential benefits must be balanced against the cost of training such persons, as well as the rate of scans requiring specialist review, in the prevailing medicolegal environment.

We raise this issue because, although the ethical duty of the physician researcher appears obvious, the appropriate management of incidental findings in research studies has not been raised in musculoskeletal imaging. It is important given the increasing use of MRI in the investigation of OA by researchers trained for the particular task but not in radiology. This issue has only recently been reviewed in the neurological literature pertaining to brain MRI and functional MRI, in which the researchers performing measurements may not have clinical or radiological training [6,8]. The prevalence of significant abnormality ranged from 18% in 1,000 healthy volunteers at the National Institutes of Health [6] to 37.2% in 129 healthy control subjects undergoing functional MRI at Stanford University (Palo Alto, CA, USA) [7] and 21% in 225 functional brain MRI scans in healthy children [16]. The prevalence of abnormalities requiring further urgent referral has been described as approximately 2% [7,8]. As a result, research protocols now incorporate detailed consent forms addressing this issue and structured mechanisms to ensure that images are appropriately reviewed [7,8]. This issue has prompted institutions to institute formal plans to deal with the ongoing management of these incidental lesions at many levels, incorporating clinical, ethical, and financial aspects. Whilst, based on these data, the prevalence of clinically important neurological lesions is likely to be higher than that of musculoskeletal abnormalities, we cannot ignore our duty of care to study participants: the physician researcher is obligated to place the well-being of the individual study participant above the interests of science or society [5].

## Conclusion

This study underscores the importance of ensuring that MRI scans performed for research purposes are also reviewed by a suitably trained specialist to ensure that lesions incidental to the purpose of the scan are not missed and receive appropriate follow-up. Collaboration with an expert MRI radiologist may facilitate the institution of an appropriate system. Although the frequency of these lesions is low, they may have clinical significance and may be the first sign of life-threatening disease. Researchers should consider incorporating radiological screening of all MRI for incidental abnormalities and appropriate medical follow-up when designing studies using this technology.

## Abbreviations

MRI = magnetic resonance imaging; OA = osteoarthritis; SD = standard deviation.

## Competing interests

The authors declare that they have no competing interests.

## Authors' contributions

RG was involved in initial study design and drafted the manuscript. SS and RO were involved in study conception and manuscript revision. SRD and PRE were involved in data acquisition and revised the manuscript. AEW conceived the study and was involved in study design, data interpretation, and manuscript drafting and revision. All authors read and approved the final manuscript.
